# Experimental Evidence That Prenatal and Postnatal Developmental Stress Affects the Adult Seminal Fluid Proteome in a Precocial Bird

**DOI:** 10.1111/mec.70257

**Published:** 2026-01-28

**Authors:** Chloe Mason, Martin Garlovsky, Oscar Vedder, Trong Khoa Pham, Rachel George, Barbara Tschirren, Nicola Hemmings

**Affiliations:** ^1^ School of Biosciences University of Sheffield Sheffield UK; ^2^ Applied Zoology, Faculty of Biology, Technische Universität Dresden Dresden Germany; ^3^ Institute of Avian Research Wilhelmshaven Germany; ^4^ biOMICS Mass Spectrometry Facility University of Sheffield Sheffield UK; ^5^ Centre for Ecology and Conservation University of Exeter Penryn UK

**Keywords:** fertility, liquid chromatography–tandem mass spectrometry, maternal investment, proteomics, seminal fluid protein composition, silver spoon effect

## Abstract

Seminal fluid proteins are important modulators of male fertility and reproductive success, yet little is known about how their abundance responds to early‐life developmental stress. Japanese quail *Coturnix japonica* ) males produce a unique seminal foam that enhances fertilisation success. We characterised the proteome of the seminal foam for the first time and assessed how its composition is influenced by prenatal and postnatal developmental stress. Proteomic identification using liquid chromatography–tandem mass spectrometry and subsequent gene ontology (GO) analysis of chicken ( *Gallus gallus* *domesticus*) orthologs suggested roles for the foam proteome in sperm maturation and DNA protection, semen liquefaction, sperm plasma membrane homeostasis and energy production for sperm motility. Males that experienced prenatal stress exhibited increased abundance of proteins involved in lipid metabolic processes, inflammation and oxidative stress, including proteolytic enzymes, interleukin receptors and avidin‐like proteins. Similarly, males that exhibited postnatal stress exhibited increased abundance of proteins involved in chromatin organisation, carbon metabolism and oxidative stress. Nine proteins involved in metabolic processes and antioxidant processes were consistently more abundant across developmentally stressed males from both experiments, suggesting convergent responses to early‐life stress. These results demonstrate that early development environments can alter the seminal foam proteome of adult males, with potential implications for ejaculate quality and fertilisation ability.

## 1 Introduction

Identifying the causes of variation in male reproductive success is essential to understanding the process of sexual selection (Andersson 1994; Birkhead 2010). Variation in sperm and testes traits influences male fertilisation success under sperm competition (Lüpold et al. 2020). However, research into the role of non‐sperm ejaculate components in post‐copulatory sexual selection has only gained momentum in recent years. In internally fertilising species, males transfer sperm along with seminal fluid comprised of somatic cells (e.g., immune cells), macromolecules (carbohydrates, fats, vitamins and minerals), hormones and proteins (Hopkins et al. 2017; Poiani 2006).

Seminal fluid proteins (SFPs) are particularly important in male and female reproduction and include antioxidants, lipases, lectins, proteases and protease inhibitors with a diverse range of functions (Chapman 2001; Avila et al. 2011; Perry et al. 2013; Ramm 2020; Santiago‐Moreno and Blesbois 2020). The function of SFPs has mainly been studied in mammals, insects and to some extent, birds, where they can promote sperm competition success through mediating sperm function (den Boer et al. 2008; Labas et al. 2015; Jodar et al. 2017; Thélie et al. 2019; Santiago‐Moreno and Blesbois 2020), storage (den Boer et al. 2009; King et al. 2011) and maturation (Manjunath and Thérien 2002; Douard et al. 2004), as well as providing immune protection (Dorus et al. 2012; Atikuzzaman et al. 2017). SFPs can also act beyond sperm traits by modifying female reproductive behaviour (Chapman et al. 2003; Liu and Kubli 2003; Bath et al. 2017), mating plug formation (Ram and Wolfner 2009; Stockley et al. 2020), promoting oviposition (Chapman et al. 2003; Liu and Kubli 2003; Goenaga et al. 2015), and modulating female immune responses (Short and Lazzaro 2010; Schjenken and Robertson 2014) and physiology (Sasanami et al. 2015; Schjenken and Robertson 2020). Furthermore, SFPs exhibit high rates of evolutionary change, resulting in between‐species divergence and within‐species variation (Chapman 2001; Ramm et al. 2009; Goenaga et al. 2015; Garlovsky et al. 2020).

Across species, there is evidence that seminal fluid production is affected by the environment. A meta‐analysis found investment in SFP production is highly sensitive to nutrient availability, particularly dietary protein intake, whilst sperm traits were only moderately affected (Macartney et al. 2019). However, how environmental factors influence the protein composition of seminal fluid, as opposed to just the amount produced, is less well understood across taxa. In *Drosophila*, the main genus in which this work has been carried out, SFP abundances have been shown to be affected by diet (Zendeer et al. 2023), male‐male competition (Hopkins et al. 2019; Ramm 2020), female‐mating status (Sirot et al. 2011) and male age (Sepil et al. 2020), but whether these effects are consistent across other taxa remains unclear.

Developmental conditions are likely to have long‐term effects on male reproductive investment, with nutritional conditions during postnatal development emerging as a particularly important factor (Edwards et al. 2019; García‐Vargas et al. 2019). In *Drosophila* for example, males reared on a nutrient‐restricted diet were typically smaller and partially compensated for lower mating success by investing in increased sperm numbers per ejaculate under competitive mating situations (De Nardo et al. 2021). In non‐competitive matings, however, males reared on poor‐quality diets transferred fewer sperm across successive matings compared to high‐quality diet males (Macartney et al. 2021), suggesting resource‐limited males are constrained in their long‐term investment in sperm traits. However, less is known about how dietary stress influences other components of the ejaculate, such as SFPs (Macartney et al. 2019). Investment in seminal fluid production is a large energetic expense (Friesen et al. 2015) and seminal fluid may deplete faster than sperm (Linklater et al. 2007; Reinhardt et al. 2011). *Drosophila* males reared at a higher population density with limited food were smaller in body size and transferred a greater proportion of SFPs during mating, possibly reflecting a response to increased risk of sperm competition (Wigby et al. 2016). Further proteomic analysis revealed that although larger males produced higher abundance of SFPs in their accessory glands, smaller males transferred greater quantities of SFPs during mating, suggesting that males exposed to postnatal developmental stress may invest more per mating (von Hellfeld et al. 2025).

Although resource demand is highest during postnatal development, the prenatal development environment provided by the mother can also have significant and long‐term effects on offspring phenotype and fitness (Mousseau and Fox 1998; Rhind et al. 2001). Prenatal developmental stress, such as malnutrition, can influence embryo growth, organ development, immune function and adult reproductive behaviour (Clark and Galef 1995; Desai and Hales 1997; Gorman and Nager 2004; Giordano et al. 2014). In sheep ( *Ovis aries* ), maternal undernutrition reduces the lifetime reproductive capacity of female offspring, while in males, it alters fetal plasma testosterone concentrations despite having no detectable effect on testis structure, potentially affecting later reproductive development (Rae et al. 2002).

Birds provide an ideal system for investigating the effects of prenatal and postnatal conditions on adult reproductive traits, as embryonic development occurs externally, allowing direct measurement of prenatal factors (Groothuis et al. 2005). Moreover, egg size and its composition (i.e., the proportion of nutrients, hormones and immunoglobulins) are known to alter offspring phenotype (Williams 1994). In zebra finches ( *Taeniopygia guttata* ), offspring of females with a poor diet prior to breeding have lower fecundity (Gorman and Nager 2004), but the effect of prenatal conditions on adult male reproductive investment, particularly the composition of SFPs, remains unexplored.

In this study, we experimentally investigate if pre‐ and postnatal developmental stress influences the proteomic composition of a unique seminal foam produced by male Japanese quail ( *Coturnix japonica* ). Species of the *Coturnix* (quail) genus produce a foam‐like substance upon ejaculation, secreted from a specialised gland known as the ‘proctodeal gland’ or ‘cloacal gland’ (Klemm et al. 1973). The foam is a viscous glycomucoprotein aerated by male cloacal muscle contractions (Seiwert and Adkins‐Regan 1998), as well as interactions with carbon dioxide and hydrogen produced by cloacal bacteria (Mohan et al. 2004). In Japanese quail, seminal foam constitutes a novel component of the quail ejaculate that is stored separately from sperm and is not mixed with semen until inside the female reproductive tract (Fujihara 1992; Klemm et al. 1973). SFPs may also be found in semen produced in the testes, epididymis and ductus deferens, as in other Galliformes (chicken *Gallus gallus* *domesticus* and turkey *Meleagris gallopavo* ) (Fujihara 1992), but these are not considered here. Male proctodeal gland size is a predictor of fertilisation success, and natural copulations with foam have higher fertilisation success compared to copulations without foam or with artificially placed foam (Ogawa et al. 1974; Cheng et al. 1989; Abuoghaba et al. 2024). Foam may extend the female's fertile period, increasing the likelihood of successful fertilisation (Singh et al. 2012; Abuoghaba et al. 2024), or improve sperm motility. Foam significantly prolongs sperm motility and increases sperm velocity in vitro, suggesting its components supply energy to sperm (Singh et al. 2011; Farooq et al. 2015). Lactate dehydrogenase is a protein found at high levels in quail seminal plasma (Buxton and Orcutt 1975), and lactate in foam may act as an energy source for sperm transport (Singh et al. 2011) as it does in the seminal fluid of mammals (Odet et al. 2011; Saeed et al. 2021). Foam also disaggregates sperm clumps, leading to more vigorous motility, possibly due to non‐protein components (Singh et al. 2011). Most studies investigating the effect of foam on male fertilisation success employ a foam removal technique which requires invasive surgery and is likely to interfere with copulation. However, Finseth et al. (2013) showed that non‐invasive foam removal in natural mating scenarios reduces male fertilisation success under sperm competition. The function of foam during sperm competition, possibly mediated through positive effects on the male's own sperm at a cost to a rival's fertility, suggests it evolved under sexual selection (Finseth et al. 2013). Although Japanese quail mating behaviour is difficult to observe in the wild, due to their cryptic nature, the species is most likely polyandrous, with high levels of sperm competition, evidenced by their frequent mate switching and forced copulations under semi‐natural conditions (Nichols 1991) and laboratory settings (Adkins‐Regan 1995), prolonged female sperm storage (Birkhead and Fletcher 1994; Beccardi, Tschirren, and Vedder 2025), and large testes relative to body size (Clulow and Jones 1982; Møller 1991).

The broader protein composition and function of quail foam have not yet been studied but could offer an interesting comparison to SFPs that are differentially produced but play a similar role in other Galliformes (Labas et al. 2015; Borziak et al. 2016; Słowińska et al. 2017). In this study, we used high‐throughput proteomics using liquid chromatography–tandem mass spectrometry (LC–MS/MS) to characterise the protein composition of the unique seminal foam of the Japanese quail for the first time, offering new insights as to its evolution and function. We then assess whether developmental stress affects the proteomic composition of seminal foam that males produce as adults, using males from two experiments that were aimed at manipulating resources available during pre‐ vs. postnatal development. We hypothesised that male investment in seminal foam would be more constrained if they developed in an environment with limited resources, and this would impact adult SFP production, with developmentally stressed males exhibiting lower abundances of SFPs, particularly those that modulate fertilisation success, than those that were not resource‐limited during development. Together, these experiments provide the first assessment of how early developmental stress affects the seminal fluid proteome in any vertebrate species, advancing our understanding of the function of this unique reproductive fluid and early‐life effects on ejaculate investment more broadly.

## 2 Materials and Methods

### 2.1 Study Population and Experimental Manipulation of Early Life Stress

#### 2.1.1 Experiment 1—Prenatal Developmental Stress

**FIGURE 1:**
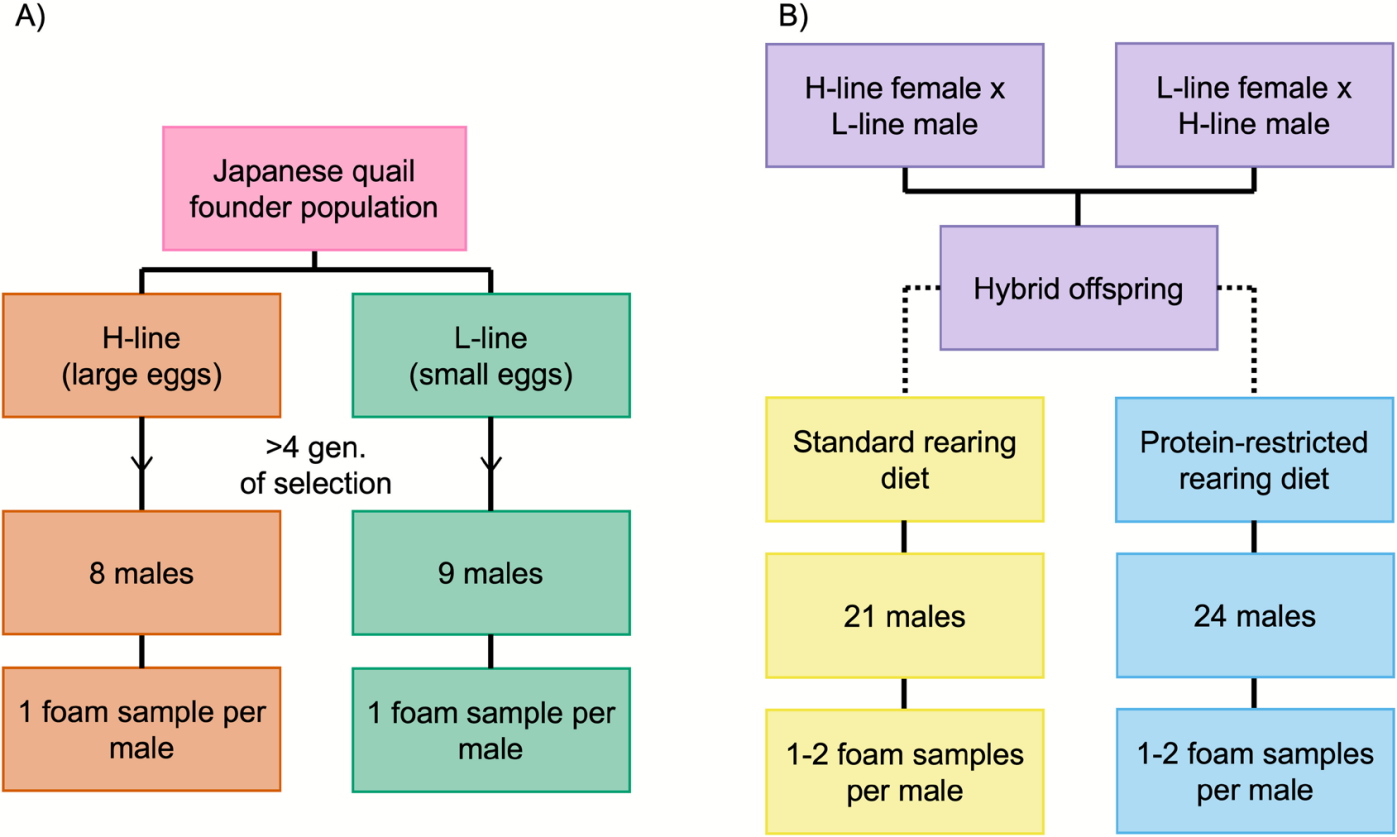
Experimental design and sample collection: (A) prenatal developmental stress (Experiment 1) and (B) postnatal developmental stress (Experiment 2).

We used artificial selection for increased and reduced maternal egg investment to manipulate prenatal developmental stress (Pick, Hutter, and Tschirren 2016). Briefly, from a founder population of Japanese quail housed in a breeding facility at the University of Zurich, Switzerland, the 10 females producing the largest and smallest eggs (relative to their body size) were assigned to the high and low maternal reproductive investment lines, respectively, in two independent biological replicates. Two sons and 2 daughters of each of the 10 females producing the largest eggs (20 sons and 20 daughters total) and 10 females producing the smallest eggs (20 sons and 20 daughters total) within their respective lines and replicates were selected for the next generation of breeding (20 breeding pairs per line). Breeding pairs consisted of unrelated males and females (not sharing any grandparents) and individuals were paired once only. See Pick, Hutter, and Tschirren (2016) for a full description of the breeding conditions. By generation four, there was a strong divergence in egg size and dried egg components (i.e., resource availability for the developing embryo) between lines (mean ± SDs: high maternal investment line = 12.46 ± 0.94 g, low maternal investment line = 11.12 ± 0.91 g; difference in absolute egg size = 1.06 standard deviations), but no difference in laying rate (Pick, Hutter, and Tschirren 2016). In 2017, individuals were transferred to the Institute of Avian Research, Wilhelmshaven, Germany, and in 2018, we sampled foam from 1‐year‐old non‐sib male offspring from the 5th and 6th generations of the selection experiment (8 males from the high maternal investment lines and 9 males from the low maternal investment lines; Figure 1A). We collected foam via cloacal and proctodeal gland massage, removing it before semen was ejaculated to avoid contamination, and stored aliquots of pure foam at −80°C until analysis.

#### 2.1.2 Experiment 2—Postnatal Developmental Stress

We used a post‐hatching food quality experiment to manipulate postnatal developmental stress (Vedder et al. 2023). In 2019, 1‐year‐old birds from the 6th and 7th generation of the selection experiment (see above) were mated with a partner from the opposite maternal investment selection line within each replicate to produce hybrid offspring. See Vedder et al. (2023) for a full description of the breeding conditions. Hatchlings were randomly distributed over rearing cages with one of two rearing diets that differed in protein content: a standard protein diet (21.0% protein, 4.0% fat and 1.1% calcium; calorific value 11.4 MJ/kg) or a protein‐restricted diet (14.5% protein, 4.0% fat and 1.0% calcium; calorific value 11.4 MJ/kg), both of which are commercially available (GoldDott, DERBY Spezialfutter GmbH, Münster, Germany). There was no difference in the size of the eggs that the hatchlings originated from between treatment groups (overall mean ± SE: 12.04 g ± 0.19; standard‐protein diet group: 12.18 g ± 0.27; protein‐restricted diet group: 11.92 g ± 0.27). The protein‐restricted diet substantially reduced growth rate without compromising chick survival, whilst the standard diet increased growth rate without leading to impairments associated with too rapid growth (Vedder et al. 2023; Vedder and Beccardi 2025). From 5 weeks onwards, birds were individually checked for cloacal foam production every 2–3 days, indicating sexual maturity (Sachs 1969). The average age for onset of sexual maturity for males was 45 days with the standard diet, and 69 days with the protein‐restricted diet. After sexual maturity, all males received a standard adult diet (19.0% protein, 4.6% fat and 4.8% calcium; calorific value 9.8 MJ/kg) (GoldDott, DERBY Spezialfutter GmbH, Münster, Germany). In 2019, we collected 1–2 foam samples per male from 21 standard rearing diet and 24 protein‐restricted rearing diet non‐sib males (Table S1; Figure 1B) while they were housed in individual cages, with the standard adult diet. The average age of males when foam was collected was 117 days ± 10. Foam samples were collected and stored as described above. In both experiments, birds were maintained on a 16:8 light: dark cycle at ~20°C and had constant access to water, grit and food. All animals were kept under licences provided by the Veterinäramt JadeWeser (permit number 42508_03122020).

### 2.2 Sample Preparation

We defrosted foam samples at room temperature for 12 h, then pooled smaller samples from the same selection lines/diet treatments within each replicate to ensure sufficient material for mass spectrometry (MS) analysis. Overall, this gave us 4 pooled replicate samples per selection line (high maternal investment line and low maternal investment line) from Experiment 1, and 12 pooled replicate samples per dietary treatment (standard‐diet and protein‐restricted diet) from Experiment 2. In Experiment 1, 4 high line samples and 3 low line samples contained foam from 2 males (1 foam sample per male), and 1 low line sample contained foam from 3 males (1 foam sample per male). In Experiment 2, we had 6 replicates from each hybrid cross type (high line mother × low line father, and low line mother × high line father). Twelve standard‐diet samples contained foam from 3 males (1 foam sample per male), and 12 protein‐restricted samples contained foam from 4 males (1 foam sample per male).

We added four times the sample volume of ice‐cold acetone (−20°C) to each sample, then vortexed and incubated them for 4 h at 20°C before centrifuging for 10 min at 13,000 rpm at 4°C to isolate foam proteins. The remaining supernatant was discarded, and we left the protein pellet at room temperature until the acetone evaporated completely.

We resuspended the protein pellet in 25 μL of protein lysis buffer (5% SDS (Sigma–Aldrich) and 100 mM TEAB (ThermoFisher), pH 8.5). We quantified the total protein concentration using a BCA assay (ThermoFisher) and normalised to 25 μg of protein per replicate for proteomic analysis. We reduced the protein by 20 mM DTT (ThermoFisher) using a thermoshaker at 95°C, 800 rpm for 10 min, and after cooling for 5 min at room temperature, we alkylated proteins using 40 Mm 2‐iodoacetamide (Sigma–Aldrich) using a thermoshaker at room temperature, 800 rpm for 30 min in the dark. We then digested proteins using a suspension trapping technique (S‐Trap) according to the manufacturer's protocol (ProtiFi). Briefly, alkylated proteins were acidified by adding 2.5 μL 12% phosphoric acid, followed by 365 μL S‐trap binding buffer (90% aqueous methanol, 0.1 M TEAB, pH 7.1). We transferred samples to S‐Trap columns gently and centrifuged for 60 s at 4000 × *g* to trap the denatured proteins.

We washed trapped proteins 5 times with 150 μL binding buffer, centrifuging between each addition, before transferring proteins to clean 1.5 mL Eppendorf tubes for protein digestion. We added 25 μL of MS grade Trypsin (ThermoFisher) in 50 mM TEAB buffer (concentration 0.1 μg/μL) to each S‐Trap and incubated at 47°C for 1.5 h without shaking to digest the proteins into peptides. Peptides were then eluted using a series of solvents: 40 μL 50 mM TEAB, 40 μL 0.2% aqueous formic acid (ThermoFisher), 40 μL 50% ACN in 0.2% formic acid and 40 μL 80% ACN in 0.2% formic acid. Samples were centrifuged at 4000 × *g* for 60 s between solvent additions. We collected eluted peptides and dried them in a vacuum concentrator (Eppendorf) for 2 h before reconstituting them in 60 μL 0.5% formic acid and withdrawing 4 μL for MS analysis.

### 2.3 Liquid Chromatography‐Tandem MS Analysis

We performed sample processing separately for each experiment. All MS proteomics analyses were performed at the bioMICS Mass Spectrometry Facility, University of Sheffield (https://www.sheffield.ac.uk/mass‐spectrometry) on an Orbitrap Exploris E480 mass spectrometer (ThermoFisher) equipped with a nanospray source, coupled to a Vanquish HPLC System (ThermoFisher). Peptides were desalted online using a Nano‐Trap Column (75 μm I.D.X 20 mm; ThermoFisher) and then separated using an EASY‐Spray column (50 cm × 50 μm I.D., PepMap C18, 2 μm particles, 10 Å pore size; ThermoFisher). We used a 100‐min gradient, starting from 3% to 20% buffer B (0.5% formic acid in 80% ACN) for 68 min, followed by a ramp‐up to 35% buffer B for 23 min, then to 99% buffer B for 1 min, and maintained at 99% buffer B for 9 min. MS was operated in positive mode with a cycle of 1 MS acquired at a resolution of 120,000, at m/z of 400. We subjected the top 20 most abundant multiply charged (2^+^ and higher) ions in a given chromatographic window to MS/MS fragmentation in the linear ion trap, with a scan range (m/z) of 375–1200, normalised AGC target of 300%, microscan 1, an FTMS target value of 1e4 and a resolution of 15,000.

### 2.4 Protein Identification and Bioinformatic Analysis

We compared protein abundances between the selection lines and rearing diet groups described above, as well as producing an overall characterisation of the Japanese quail foam proteome using data from all samples combined. We analysed all MS data with MaxQuant (v.1.6.10.43) and searched data against the *Coturnix japonica* protein database consisting of 27,875 proteins (Uniprot proteome ID: UP000694412) with the following search parameters: trypsin/P (2 missed cleavages) as the enzyme, methionine oxidation and N‐terminal protein acetylation as variable modifications and cysteine carbamidomethylation as a fixed modification. We set FDR for both peptides and proteins to 1% using target‐decoy approaches. Since using a two unique peptide requirement can reduce proteome coverage without substantially improving identification confidence when a robust FDR control is used, we used a ≥ 1 unique peptide cutoff.

We loaded the MaxQuant output into Perseus (v1.5.6.0) for downstream data analysis, including filtering, normalisation and statistics (Cox and Mann 2008; Cox et al. 2014). We set all label‐free quantification (LFQ) intensities as main columns, filtered the matrix to remove potential contaminant proteins and reverse sequences, then transformed LFQ intensities using the log_2_(*x*) function. For the selection treatment, we categorically annotated rows with either H (high maternal investment) or L (low maternal investment), and filtered proteins to identify those present in at least 3 replicates in at least one of the selection lines. For the diet treatment, we categorically annotated rows with either SD (standard diet) or PR (protein‐restricted diet), and filtered proteins to identify those present in at least 3 replicates in at least one of the diet groups. We examined the quality of replicates using Pearson's correlations and performed Principal Component Analysis (PCA) on the log_2_‐transfromed LFQ intensities to assess sample clustering.

We normalised each protein's (log_2_) intensity in each sample to its median value and missing values were imputed from a normal distribution (Experiment 1: 472 values imputed, 30% and Experiment 2: 1521 values imputed, 56%). We calculated log_2_ fold‐changes to describe differences between maternal investment selection lines or diet groups within each experiment. We then evaluated differences in protein abundance between selection lines or treatment groups for statistical significance (*p* < 0.05) using unpaired two‐sided Student's *t*‐tests with Benjamini‐Hochberg correction for multiple testing (FDR) on normally distributed data.

### 2.5 Gene Ontology (GO) Analysis

As the *C. japonica* genome has not been fully annotated, we identified chicken ( *Gallus gallus* *domesticu*s) orthologs using OrthoFinder v.3.0 (Emms and Kelly 2019) and the chicken reference proteome (UniProt Proteome ID: UP000000539). We assessed the overlap between the quail foam proteome, the seminal fluid proteome of the red junglefowl ( *Gallus gallus* ; Borziak et al. 2016) and domestic chicken (Labas et al. 2015), and the chicken spermatozoa proteome (Labas et al. 2015). We used the list of orthologs that were differentially abundant in a selection line or diet group for GO analysis to identify functional differences in the foam's proteome between treatments.

We performed GO enrichment analyses using the website version of DAVID v.2023q4 (Huang et al. 2009; Sherman et al. 2022). We uploaded the list of proteins that were differentially abundant within a selection line or diet group to DAVID (https://david.ncifcrf.gov/tools.jsp) and used the total seminal foam proteome as the background. In instances where a quail protein had multiple orthologs in chicken, we selected one representative ortholog for GO analysis. We downloaded the outputs for all three GO categories (biological processes, cellular components and molecular functions) including their associated statistical values, as well as the output table from the Functional Annotation Clustering Tool that clusters redundant annotation terms to identify biological themes associated with proteomes. Figures were created in R v.4.4.2 (R Core Team 2024).

## 3 Results

### 3.1 Effect of Prenatal Developmental Stress on the Seminal Foam Proteome

**FIGURE 2:**
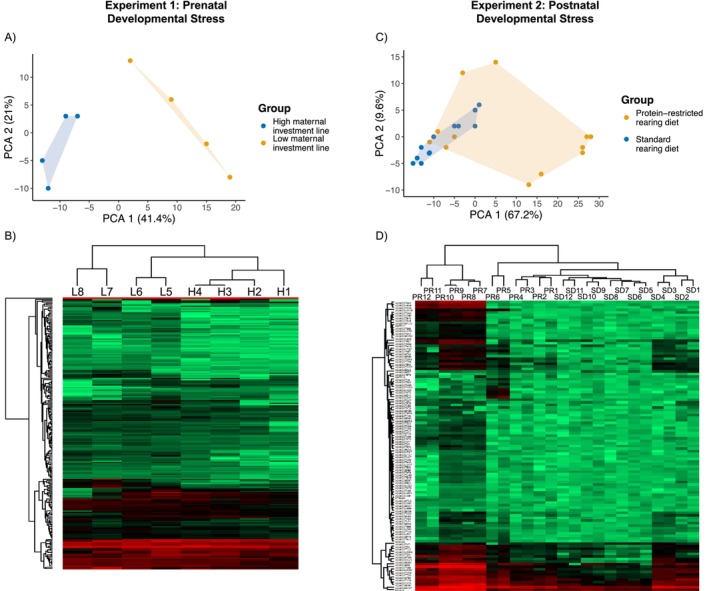
Proteomic profiles of foam samples from both experiments. Experiment 1: (A) principal component analysis (PCA) plot of protein abundance of samples from high (blue) and low (orange) maternal investment lines along PC1 (41.4%) and PC2 (21%), and (B) heat map showing the abundance of 196 detected seminal foam proteins. Rows represent individual proteins and columns represent replicate samples from high (H) and low (L) maternal investment lines. Experiment 2: (C) principal component analysis (PCA) plot of protein abundance of samples from standard (blue) and protein‐restricted (orange) rearing diet groups along PC1 (67.2%) and PC2 (9.6%) and (D) heat map showing the abundance of 103 detected seminal foam proteins. Rows represent individual proteins and columns represent replicate samples from standard rearing diet (SD) and protein‐restricted rearing diet (PRD) groups.

In the prenatal stress experiment, we identified 425 proteins across 17 Japanese quail foam samples (pooled into 4 replicates per treatment; Table S2). Of these, 196 (46.2%) were identified in at least 3 replicates within a selection line and included in the final dataset (Table S3). The relative protein abundances were strongly correlated between replicates (mean Pearson's correlation coefficient *r* = 0.86, range = 0.69–0.96). A principal component analysis showed PC1 explained 41.1% and PC2 explained 21% of the variation in the data and revealed a clear separation between males from the high and low maternal investment lines (Figure 2A,B).

**FIGURE 3:**
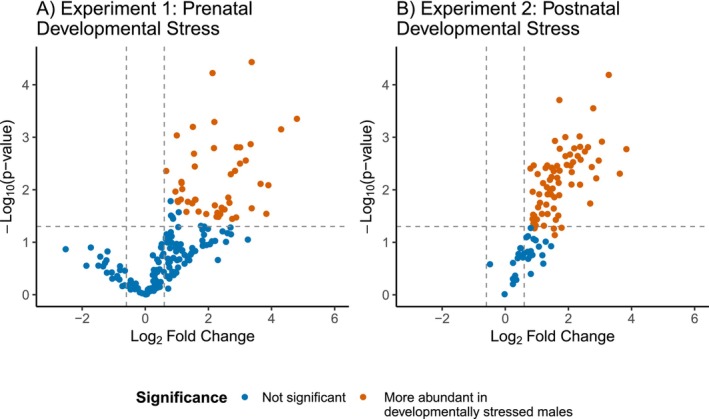
Effect of developmental stress on the quail seminal foam proteome: (A) Volcano plot of protein abundance comparing low vs. high maternal investment lines (two‐sample *t*‐test, *n* = 4 biological replicates per selection line). Orange indicates 48 of 196 proteins were more abundant in the low maternal investment lines. No proteins were more abundant in the high maternal investment lines. Blue proteins had no significant change in abundance between the high and low maternal investment lines. (B) Volcano plot of protein abundance comparing protein‐restricted vs. standard rearing diet groups (two‐sample *t*‐test, *n* = 12 biological replicates per diet treatment). Orange indicates 72 of 103 proteins were more abundant in the protein‐restricted diet group. No proteins were more abundant in the standard diet group. Blue proteins had no significant change in abundance between the standard diet and protein‐restricted diet group.

We found 48 proteins that were significantly more abundant in foam of males from the low maternal investment lines compared to males from the high maternal investment lines (for each protein log_2_ fold change > 1.5, *p* < 0.05; Table S3; Figure 3A) and 148 proteins that were present in similar quantities in both the high and low maternal investment lines (*p* > 0.05). No proteins were significantly more abundant in the high maternal investment line compared to the low maternal investment line (log_2_ fold change < 1.5, *p* < 0.05). See Table S3 for results of the two‐sided Student's *t*‐tests.

The 48 proteins that were more abundant in foam samples from the low maternal investment line 47 had chicken orthologs, and 17 had more than one (106 orthologs in total; see Table S3 for a complete list). Of these proteins, 46 (96%) had GO annotations. Our GO analysis showed that proteins that were more abundant in the low maternal investment lines were associated with lipid metabolic processes (see Table S4 for a complete list of GO terms associated with proteins that were more abundant in the low maternal investment line and associated *p*‐values). No annotation clusters were enriched in proteins that were more abundant in the low maternal investment line. See Table S5 for all annotation clusters, associated terms and *p*‐values.

### 3.2 Effect of Postnatal Developmental Stress on the Seminal Foam Proteome

In the postnatal stress experiment, we identified 401 proteins across 79 Japanese quail foam samples (pooled into 12 replicates per treatment; Table S2). Of these, 103 (25.7%) were identified in at least 3 replicates within a treatment group and included in the final dataset (Table S6). The relative protein abundances were strongly correlated between replicates (mean Pearson's correlation coefficient = 0.83, range = 0.55–0.99). A principal component analysis showed PC1 explained 67.2% and PC2 explained 9.6% of the variation in the data (Figure 2C,D).

We found 72 proteins that were significantly more abundant in foam samples from males raised on a protein‐restricted diet compared to a standard diet (for each protein log_2_ fold change > 1.5, *p* < 0.05; Table S6; Figure 3B) and 31 proteins that were present in similar quantities in foam from the standard and protein‐restricted diet males (*p* > 0.05). No proteins were significantly more abundant in foam from males fed the standard diet compared to the protein‐restricted diet (log_2_ fold change < 1.5, *p* < 0.05). See Table S6 for results of the two‐sided Student's *t*‐tests.

**FIGURE 4:**
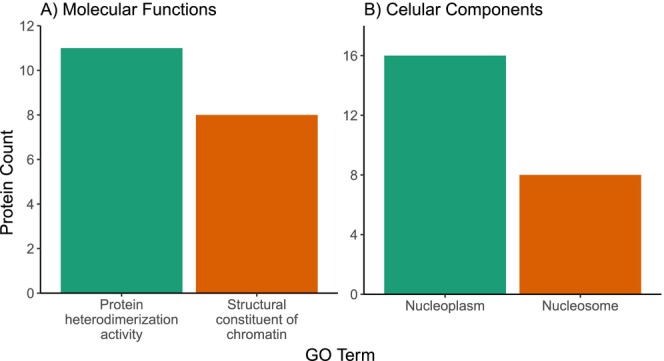
GO analysis of the proteins with increased abundance in the protein‐restricted diet group: (A) 2 molecular functions, and (B) 2 cellular components all enriched in proteins that were significantly more abundant in Japanese quail seminal foam of males fed a protein‐restricted diet compared to a standard diet. Only functional enrichment groups with *p*‐values < 0.05 are shown.

Of the 72 proteins that were more abundant in foam samples from males fed the protein‐restricted diet, 71 had chicken orthologs and 11 had more than one (128 orthologs in total; see Table S6 for a complete list). Of these proteins, 69 (96%) had GO annotations. Our GO analysis found that proteins that were more abundant in the protein‐restricted diet group were associated with nucleosome assembly, the molecular functions: protein heterodimerisation activity and structural constituent of chromatin (Figure 4A); and the cellular components: nucleoplasm and nucleosome (Figure 4B; see Table S7 for a complete list of GO terms associated with proteins that were more abundant in the protein‐restricted diet group and associated *p*‐values).

Using the Functional Annotation Clustering Tool in DAVID (Huang et al. 2009; Sherman et al. 2022), we identified that the annotation cluster histone modification and chromatin structure was enriched in proteins that were more abundant in the protein‐restricted diet group. See Table S8 for all annotation clusters, associated terms and *p*‐values.

### 3.3 Overall Characterisation of the Japanese Quail Seminal Foam Proteome

Finally, we combined data across both experiments to provide an overall characterisation of the seminal foam proteome. We identified 608 foam proteins across the two cohorts, of which 224 were identified in at least 3 replicates in a single cohort (36.8%) (Table S2). Relative protein abundances were strongly correlated between replicates (mean Pearson's correlation coefficient = 0.85, range = 0.55–0.99). See Table S9 for the 20 most abundant proteins in the foam.

Genes encoding foam proteins were highly conserved, with 96.4% of proteins (216/224) having chicken orthologs (Table S2) compared to 82.0% of all Japanese quail proteins (22,858/27,875; *χ* ^2^ = 31.17, df = 1, *p* < 0.001). We found more than one chicken ortholog for 27.7% (62/224) of quail proteins (Figure S1), and 449 chicken protein orthologs in total, which are encoded by 266 distinct chicken genes. See Table S10 for full ortholog protein list.

Eight foam proteins (4%) did not have chicken orthologs (Table S11) and we inferred their functions in UniProt (The UniProt Consortium 2025). Four were predicted to be isoforms of neuroblast differentiation‐associated protein AHNAK‐like and associated with the nucleus and regulation of RNA splicing. Two predicted Ig‐like domain‐containing proteins may be associated with an immunoglobulin‐mediated immune response. We found aldo‐keto reductase family 1 member B1 (aldose reductase) in foam, inferred from homology, and finally, we found a predicted secreted protein associated with the transmembrane helix with no known function.

**FIGURE 5:**
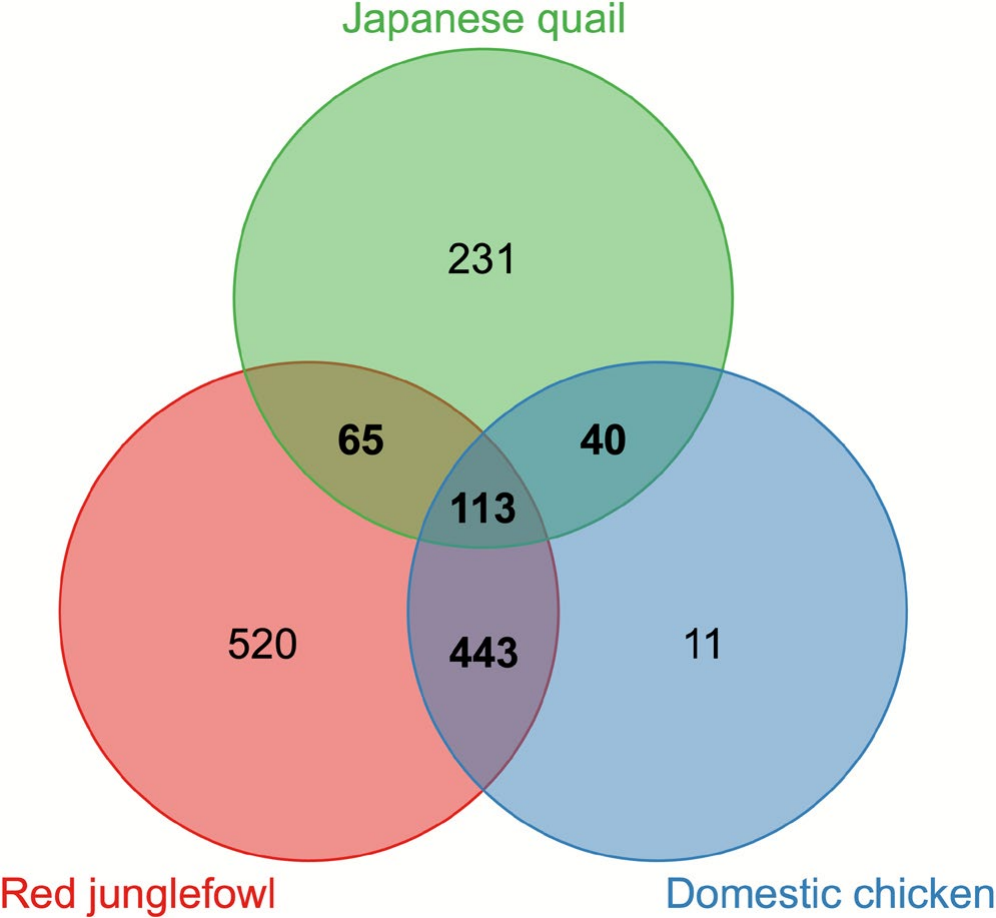
Venn diagram indicating the size and overlap between the seminal foam proteomes of the Japanese quail ( *Coturnix japonica* ), red junglefowl ( *Gallus gallus* ) and domestic chicken (*Gallus gallus domesticus*).

Comparing proteomes, we found that 7.6% (34/449) and 14.7% (66/449) of chicken orthologs identified in quail foam were also in domestic chicken and red junglefowl seminal fluid, respectively, and 34.1% (153/449) and 39.6% (178/449) of foam proteins had isoforms found in chicken and red junglefowl seminal fluid, respectively (Figure 5; Table S10). Comparing genomes, we found 31.6% (84/266) and 40.2% (107/266) of genes encoding quail foam proteins encoded SFPs in chicken and red junglefowl seminal fluid, respectively (Table S10). There was evidence that proteins found in chicken sperm were also present in quail foam, with 29.2% (131/449) of foam protein having isoforms identified in chicken spermatozoa (Table S10). Of these, 48 were not found in chicken seminal fluid. The remaining 231 quail foam proteins were not found in either chicken or red junglefowl seminal fluid. GO analysis of proteins unique to quail foam revealed a significant association with proteolysis (Benjamini‐Hochberg corrected *p* = 4.5E‐6, FDR = 4.5E‐6).

**FIGURE 6:**
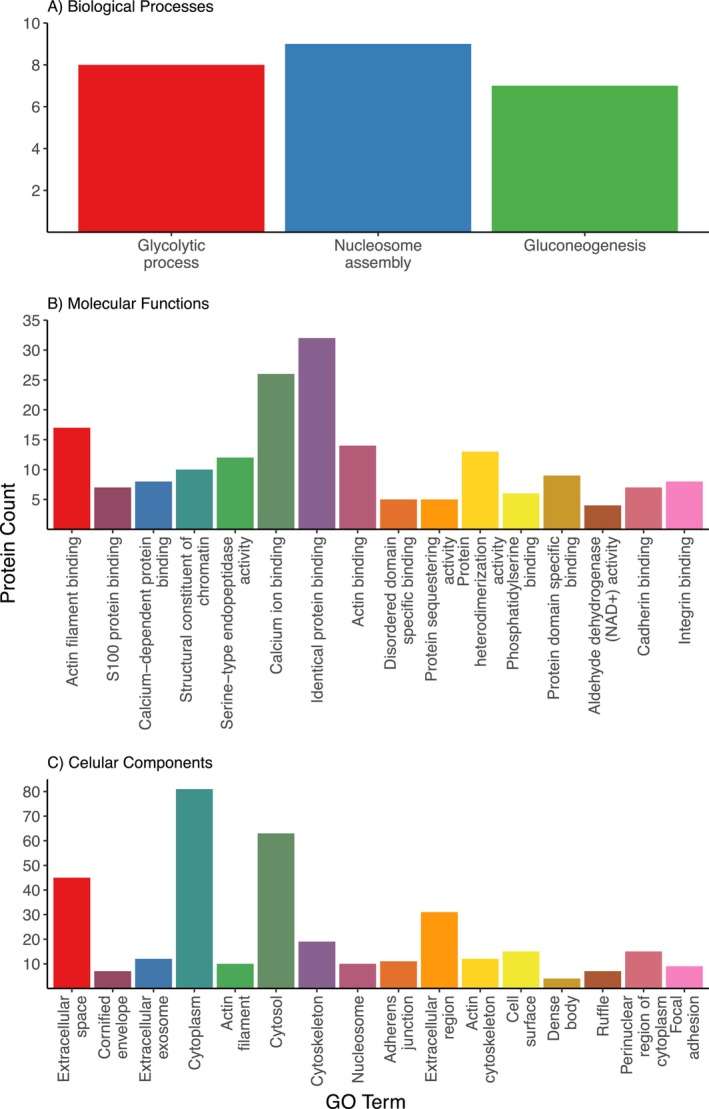
GO analysis of the Japanese quail ( *Coturnix japonica* ) seminal foam proteome: (A) 7 biological processes, (B) 18 molecular functions and (C) 15 cellular components significantly enriched in the Japanese quail foam proteome, as identified by DAVID. Only functional enrichment groups with Benjamini‐Hochberg corrected *p*‐values < 0.01 and passing a 1% false discovery rate threshold are shown.

GO analysis of chicken orthologs using the *G. gallus* gene list as the background identified 3 biological process terms (Figure 6A), 16 molecular functions (Figure 6B) and 16 cellular components (Figure 6C) significantly enriched in quail foam (see Table S12 for all GO terms and associated *p*‐values), together suggesting the foam plays a role in sperm maturation and motility, proteolysis, protein binding and immune regulation. The Functional Annotation Clustering tool in DAVID (Huang et al. 2009; Sherman et al. 2022) identified 35 clusters (Table S13), 17 of which were significantly enriched in the foam proteome (Table S14). As a relatively high number of proteins were associated with proteolysis, these proteins were identified individually and assigned to their appropriate protease class (Table S15).

### 3.4 Overlaps in Proteomes

**FIGURE 7:**
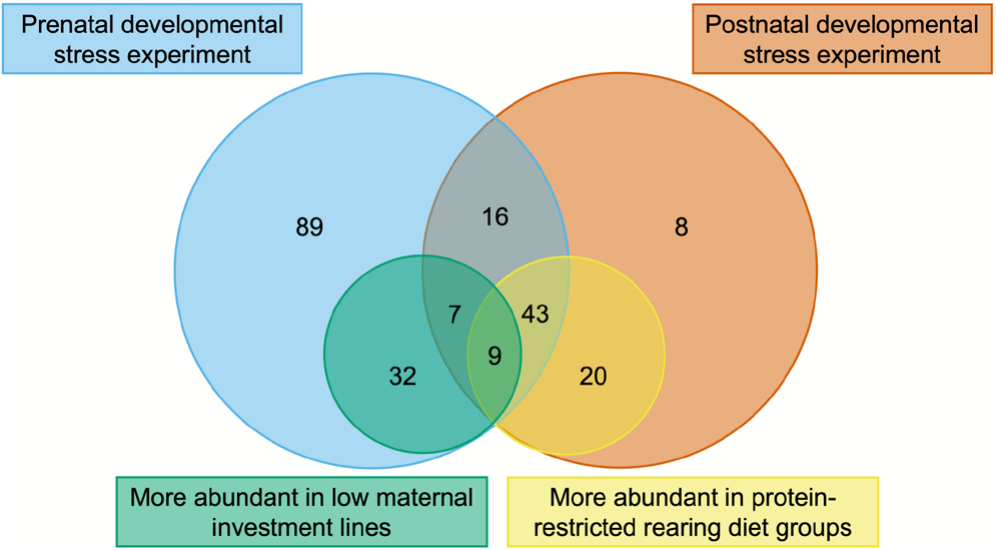
The number of proteins differentially expressed between the prenatal and postnatal developmental stress experiments, and proteins that were significantly more abundant within treatments in both experiments. The number of the same overlapping proteins between experiments and treatments are shown.

Nine proteins that were more abundant in foam samples from the low maternal investment line in Experiment 1 were also more abundant in foam of the protein‐restricted diet group in Experiment 2 (Figure 7). Of these, 6 were associated with metabolic pathways (Table 1). Their functions were inferred using the UniProt database: alpha‐amylase is an enzyme involved in glucose production (Zakowski and Bruns 1985). Transketolase catalyses the reversible conversion of sugar phosphates into glycolytic intermediates, providing a link between the non‐oxidative pentose phosphate shunt and glycolysis (Zhao and Zhong 2009). Malate dehydrogenase plays a central role in aerobic cellular respiration, facilitating ATP generation from glucose (Wolyniak et al. 2024), whilst ATP synthase drives ATP production from ADP during oxidative phosphorylation (Neupane et al. 2019). Aldo‐keto reductase family 1 member B10 detoxifies reactive carbonyl compounds in cells, and aldehyde dehydrogenase 1 family member A1 detoxifies aldehydes, both protecting cells from oxidative stress and cellular death (Shortall et al. 2021; Wang et al. 2009). Although many of these proteins are involved in metabolic pathways, no biological process, molecular function or cellular component GO terms were significantly enriched after multiple testing correction, as expected with a small number of proteins.

**TABLE 1:** Quail proteins and their chicken orthologs that were significantly more abundant in males exposed to stressful pre‐ and postnatal developmental conditions, along with their annotations from the Functional Annotation Chart in DAVID (Huang et al. 2009; Sherman et al. 2022).

Quail protein Uniprot AC	Chicken ortholog Uniprot AC	Chicken ortholog protein name	Chicken ortholog gene symbol	Functional annotation
A0A8C2T207	A0A8V0Z6F8	Mesothelin‐like protein	MSLNL	
A0A8C2U7G7	A0A8V0YKA1	Aldo‐keto reductase family 1 member B10	AKR1B10	Metabolic pathway, cytosol
A0A8C2SWD0	A0A1D5PFB8	Dynactin subunit 2	DCTN2	Cytosol
A0A8C2UFC7	Q98942	Alpha‐amylase	amy	Metabolic pathway
A0A8C2TLF1	F1P1A5	Transketolase	TKTL1	Metabolic pathway, cytosol
A0A8C2TH69	E1BVT3	Malate dehydrogenase	MDH2	Metabolic pathway
A0A8C2TV59	A0A1D5PE96	Plastin‐3	PLS3	Cytosol
A0A8C2T9A2	F1NJC7	Aldehyde dehydrogenase 1 family member A1	ALDH1A1	Metabolic pathway, cytosol
A0A8C2SZ10	A0A1D5PN54	ATP synthase subunit alpha	ATP5F1AZ	Metabolic pathway

## 4 Discussion

Here, we characterised the proteome of a unique reproductive foam produced by male Japanese quail and tested how it is influenced by developmental stress. We identified a total of 224 proteins in the foam, of which over 96% have orthologs in the closely related chicken, and at least 48% have been identified in chicken seminal fluid. Our interrogation of the foam proteome suggests roles in sperm maturation and DNA protection, semen liquefaction, sperm plasma membrane homeostasis and energy production for sperm motility.

We found that both pre‐ and postnatal developmental stress can affect reproductive traits in adult male Japanese quail by altering the protein composition of seminal foam. First, using lines selected for divergent maternal investment (measured as egg size relative to female body size), we found that males from the low maternal investment lines, which developed in relatively small, less‐provisioned eggs, exhibited increased abundance of 48 proteins in their seminal foam. These proteins were primarily associated with lipid metabolic processes. Second, to measure an alternative axis of early developmental stress, we compared males that received different quality diets post‐hatching and found that males that received a protein‐restricted rearing diet exhibited increased abundance of 72 proteins. These proteins were primarily associated with nucleosome assembly and chromatin structure. Notably, nine proteins were consistently more abundant in foam samples from both the low maternal investment line and the protein‐restricted males. These proteins were primarily associated with metabolic pathways, suggesting a convergent molecular response to early developmental stress across pre‐ and post‐hatching stages.

### 4.1 Prenatal Developmental Stress and Impaired Reproductive Function

Several proteins that were more abundant in foam of males from the low maternal investment lines (Experiment 1) suggest physiological dysregulation associated with oxidative stress, inflammation and impaired reproductive function. The low maternal investment lines used in this study have previously been shown to have lower fertilisation success compared to males of the high maternal investment lines (Pick et al. 2017), however, few differences in sperm form or function have been detected between the lines (Mason et al. 2024). Differences in foam composition may therefore go some way to explaining their different fertilising potential between investment lines.

Several proteins identified in the foam were associated with lipid metabolic processes, indicating that lipid regulation may be an important component of seminal fluid composition in males with reduced fertilisation success. Lipid‐modifying enzymes and lipid binding proteins, including pancreatic lipase‐related proteins, can influence membrane stability, energy availability and the biochemical conditions encountered by sperm (Sias et al. 2005; Furse et al. 2022; Tao et al. 2023). Differences in the lipid metabolic activity between males from high and low maternal investment lines may reflect altered physiological pathways associated with reduced fertilisation success. Abnormal lipid homeostasis can cause spermatogenic dysfunction and consequential infertility (Davis 1980; Lu et al. 2016; Thankamoni et al. 2024), with elevated lipid levels further promoting reactive oxygen species production (Agarwal et al. 2014). In low maternal investment line males, the increased abundance of lipid metabolic enzymes may impair sperm function during fertilisation. These differences in lipid metabolic activity provide useful context for interpreting other molecular differences between selection lines, including those linked to oxidative stress.

Several proteins that were more abundant in foam from low maternal investment line males are biomarkers of oxidative stress. For example, fructose‐bisphosphate aldolase is an enzyme that catalyses a key step in glycolysis (Bhagavan and Ha 2015) and is overexpressed in human seminal ejaculates with high levels of oxidative stress (Sharma et al. 2013). Additionally, alpha‐enolase is used as a biomarker of reduced semen quality in several species including humans (Force et al. 2002; He et al. 2014) and mallards ( *Anas platyrhynchos* ) (Tang et al. 2022), and increased expression may over‐activate the apoptosis signal pathway, resulting in disturbances during spermatogenesis (Xiong et al. 2022). These enzymes, when present in high concentrations, could signal increased oxidative stress and impaired sperm maturation in adult males that experienced prenatal developmental stress, with potential consequences for fertilisation. While oxidative stress in females does not differ between the lines (measured as reactive oxygen species production in blood plasma) (Pick, Hutter, Ebneter, et al. 2016), females from the low maternal investment lines (but not high maternal investment lines) exhibited a marked increase in oxidative stress under breeding conditions (Pick, Hutter, Ebneter, et al. 2016). It is therefore possible that both males and females from the low maternal investment lines are susceptible to oxidative stress, and this may be a consequence of prenatal developmental stress or a correlated response to relaxed selection on maternal investment.

Increased abundance of avidin and interleukin receptors in the foam from low maternal investment line males provides evidence of inflammation in reproductive tissue. Interleukins are cytokines involved in immune cell regulation and inflammation modulation (Verkhratskiĭ 2023). White blood cells, which produce interleukins, are often in higher abundance in semen of humans with genital tract inflammation and fertility issues (Barratt et al. 1990; Sharma et al. 2013). Interleukins are activated by binding to interleukin receptors (Martin and Falk 1997) and interleukin receptor accessory proteins (Martin and Falk 1997; Casadio et al. 2001). Specifically, interleukin 1 receptor accessory protein (IL1RAP) induces multiple physiological responses to inflammation, infection and tissue damage (Dinarello 1996). Additionally, avidin‐related proteins are known to increase in response to infection, tissue injury and inflammatory stress (Board and Fuller 1974; Kunnas et al. 1993) and bind to biotin in bacteria to prevent bacterial growth as part of the immune defence (Dillon 2014). Avidin is linked to promoting sperm activation and longevity in the female reproductive tract of turkeys ( *Meleagris gallopavo* *domesticus*) (Foye‐Jackson et al. 2011) as well as protecting developing embryos against microbial infections in the female oviduct (Korenman and O'Malley 1968; Board and Fuller 1974). The increased abundance of the immune proteins suggests that males that undergo prenatal developmental stress may face inflammation as adults.

Males from the low maternal investment line exhibited increased abundance of proteases, including higher levels of trypsin and pancreatic elastase II, without corresponding increased abundance of protease inhibitors. Numerous proteases found in seminal fluid across taxa regulate multiple downstream activities involved in immunity, cell cycle regulation and tissue morphogenesis via the hydrolysation of peptide bonds (Ram and Wolfner 2009; Laflamme et al. 2014). However, these proteases require tight control by protease inhibitors to prevent premature activation of pathways or tissue damage (Laflamme and Wolfner 2013). In human seminal fluid, pancreatic elastase II plays a role in elastin hydrolysis, and increased elastase levels are associated with increased white blood cells, bacterial infection and inflammation in infertile males (Zorn et al. 2003). Whilst increased elastase levels are negatively correlated with semen volume, they have limited effects on sperm characteristics. Instead, elastase may damage the female reproductive tract, preventing fertilisation (Zorn et al. 2000). Furthermore, trypsin‐like proteases have diverse roles, including induction of egg‐laying in insects (Marshall et al. 2009) and mediation of sperm function and fertility across taxa (Green and Summers 1980; Inaba et al. 1993; Friedländer et al. 2001; Kodama et al. 2002; Miyata et al. 2012; Stephens et al. 2018). In mammals, increased trypsin concentrations lead to more rapid liquefaction of viscous semen and increased motility, which is a possible function of the quail foam (Singh et al. 2011), but at too high a concentration can lead to sperm fragmentation and compromised sperm function (Marson et al. 1988; Flores‐Herrera et al. 2012). The elevated abundance of these proteases, without corresponding regulatory proteins, supports the hypothesis that low maternal investment line males are susceptible to inflammation and impaired reproductive function, either through sperm fragmentation or by altering the environment of the female reproductive tract.

Given the limited evidence for reproductive costs of oxidative stress in birds (Speakman and Garratt 2014; Beccardi, Salmón, and Vedder 2025), defining reliable markers of oxidative stress and inflammation is essential for clarifying how early developmental stress influences adult male ejaculate quality. Individuals that experience prenatal developmental stress may exhibit hidden costs in their ejaculate composition, with metabolic and immune protein expression reflecting stress responses rather than enhanced functional investment. Increased SFP abundance may indicate constrained or dysregulated reproductive physiology, consistent with the reduced fertilisation success reported for the low maternal investment selection lines in this system (Pick et al. 2017). Previous work demonstrated that high investment line males caused greater oxidative damage in females following mating (Romero‐Haro et al. 2023), highlighting that male oxidative condition and female‐post mating oxidative costs are decoupled.

### 4.2 Postnatal Dietary Protein Restriction and Its Impact on Seminal Fluid Function

Similar to males that experienced pre‐natal developmental stress, our proteomic analyses identified several proteins that were more abundant in males raised on a post‐hatching protein‐restricted diet (Experiment 2), which may indicate oxidative stress and reduced semen quality. For example, glyceraldehyde‐3‐phosphate dehydrogenase (GAPDH) abundance increases in response to heat shock and oxidative stress in human sperm (Sharma et al. 2013) and is higher in abundance in chicken ( *G. gallus* *domesticus*) semen with reduced sperm motility (Li et al. 2020). Additionally, triosephosphate isomerase is involved in the sperm acrosome reaction and binding of sperm to the egg's zona‐pellucida (Auer et al. 2004), and its increased abundance indicates reduced sperm quality in boar ( *Sus domesticus* ) and human seminal fluid (Siva et al. 2010; Vilagran et al. 2016), and reduced fertility in bulls ( *Bos taurus* ) (Soggiu et al. 2013).

Males that were protein‐restricted during development showed an increased abundance of histones involved in protein heterodimerisation activity and chromatin organisation in the nucleus, potentially indicating inflammation (Singh et al. 2025). Elevated extracellular histone levels in biofluids are also associated with inflammation, infection and tissue damage (Singh et al. 2022, 2025). Specifically, histone H4 can affect sperm morphology and motility (Schon et al. 2019) and impaired histone‐protamine exchange during spermatogenesis can reduce fertilisation (Carrell et al. 2007). The function of extra‐nuclear histones, particularly in seminal fluids, and their effect on reproduction is an ongoing area of research. Additionally, immunoglobulin lambda‐like polypeptide 1 (IGLL1) is a type of immunoglobulin free light chain which has been associated with inflammation and reduced sperm quality in humans (Basile et al. 2022; Bruno et al. 2022). In other biofluids, lambda light chains are responsible for activating inflammatory responses in autoimmune disorders (Napodano et al. 2019). Our data provide the first evidence that IGLL1 originates from a reproductive accessory gland and is modulated by diet early in life. Overall, this provides evidence that post‐natal developmental stress can have long term effects on reproductive accessory gland inflammation status in adults, with potential negative consequences for sperm quality.

The increased abundance of glycolytic enzymes (such as pyruvate kinase and malate dehydrogenase), S100 calcium ion binding proteins and cytoskeletal proteins (such as tropomyosin, myosin, actin, spectrin and desmoplakin) in the foam of males raised on a protein‐restricted diet may reflect compensatory mechanisms to preserve reproductive function (Donato et al. 2013). In birds, glycolysis is an important energy‐generating pathway for sperm motility (Froman and Kirby 2005). Protein‐restricted males may rely more greatly on glycolysis to compensate for impaired sperm mitochondrial function (Ford 2006). Furthermore, S100 proteins are known to regulate calcium‐dependent cellular processes including energy metabolism, cytoskeletal organisation and cell survival, and may serve a protective role by supporting sperm function in the female reproductive tract (Donato 2001; Sakaguchi et al. 2008; Leśniak et al. 2009; Donato et al. 2009). S100 proteins may also promote immune tolerance in the female reproductive tract, preventing the female's immune system attacking foreign sperm cells (Sorci et al. 2011; Schjenken and Robertson 2020), but see Słomnicki et al. (2009). These findings suggest developmentally stressed males invest in the maintenance and survival of sperm, and in the literature, quail show preservation of sperm quality under dietary‐protein restriction (Arscott and Parker 1963; Retes et al. 2019; Tyler et al. 2021). However, developmental stress may elicit physiological trade‐offs, balancing immediate reproductive performance against potential long‐term fitness costs. For example, in *Drosophila*, males have been shown to buffer ejaculates against adverse conditions by transferring higher abundances of SFPs and investing more in each mating opportunity, potentially as a response to fewer mating opportunities overall (von Hellfeld et al. 2025).

Dietary‐protein availability is a major determinant of oxidative balance, as amino acids underpin the synthesis of antioxidants (Egbujor et al. 2024). In birds specifically, protein restriction during development can increase oxidative stress by disrupting the production and function of antioxidants, leading to an increase in reactive oxygen species (Faraguna et al. 2025). Whilst some reactive oxygen species are required for reproduction, an imbalance between reactive oxygen species and the capacity of antioxidant mechanisms results in oxidative stress (Costantini 2008). Oxidative stress may lead to alterations in protein expression in sperm and seminal fluid, accelerating functional decline by promoting lipid membrane peroxidation, alkylation of proteins associated with mitochondrial function and flagellar movement, and loss of motility and membrane integrity (Aitken et al. 2012; Moazamian et al. 2015). Damage to sperm morphology and DNA integrity impairs fertilisation (Azenabor et al. 2015; Dada 2017; Agarwal et al. 2018; Barati et al. 2020; Candela et al. 2021). Rather than reflecting increased reproductive investment, an increased abundance of metabolic enzymes and oxidative stress biomarkers in protein‐restricted males may represent compensatory mechanisms that sustain sperm performance under oxidative stress. We show that protein intake during early‐life development can have long‐term effects on the proteome with potential consequences for oxidative balance.

### 4.3 Metabolic Adaptations to Early Developmental Stress

Adult males exposed to early developmental stress, either pre‐ or postnatally, exhibited increased abundance of seminal foam proteins associated with metabolic pathways, particularly glycolysis, suggesting a conserved response. Malate dehydrogenase and alpha‐amylase, both involved in glycolysis, may support energy production and thereby sperm function. In mammalian seminal fluid, alpha‐amylase reduces sperm viscosity (Bunge and Sherman 1954; Mendeluk et al. 2000). In red junglefowl ( *G. gallus* ) seminal fluid, alpha‐amylase is present at low abundance and does not contribute to sperm performance (Borziak et al. 2016), but it may play a more important role in quail semen (Buxton and Orcutt 1975). ATP synthase may also contribute to energy homeostasis by producing extracellular ATP (Guo et al. 2019). Since both treatment groups also exhibited biomarkers of oxidative stress, it is possible developmental stress can have generalised long‐term physiological consequences on male fertility, even when no longer exposed to stress as an adult (Breitbart et al. 2005; Xiao and Yang 2007), and the increased abundance of glycolytic enzymes may be an adaptive response to maintain sperm function and fertility. Alternatively, increased metabolic activity could contribute to oxidative stress, suggesting a trade‐off between compensatory metabolism and redox imbalance (Speakman and Garratt 2014).

Several proteins that were more abundant in developmentally stressed males are involved in oxidative stress responses and may mitigate sperm against oxidative damage. Transketolase contributes to the production of NADPH which balances reactive oxygen species and maintains mitochondrial membrane potential (Perl et al. 2006) and is critical for ATP synthesis and sperm cell survival. Impaired function of transketolase may lead to oxidative damage and male infertility (Li et al. 2013; Perl 2007). Aldo‐keto reductase B10 (AKR1B10) and aldehyde dehydrogenase A1 detoxify reactive carbonyl and aldehyde compounds that are produced under oxidative stress (Wang et al. 2009; Shortall et al. 2021). Whilst AKR enzymes are found in male mammalian reproductive tissue (Kobayashi et al. 2002; Iuchi et al. 2004), the physiological function of other AKR enzymes other than AKR1B1 remains poorly characterised. Detoxification of reactive oxygen species may help preserve fertility under developmental stress (Gibb et al. 2016), consistent with adaptive increases in antioxidant activity seen across taxa during oxidative stress (Faraguna et al. 2025). Future research into trade‐offs between reproductive investment and other physiological functions, such as immune regulation (Knowles et al. 2009) and brain size (Kotrschal et al. 2013), in developmentally stressed males will be a fruitful area for further investigation.

Differences in the number of proteins identified between Experiment 1 and Experiment 2, as well as the relatively low overlap in proteins detected (33%), may be due to sampling limitations. Pooled samples were used to ensure sufficient material for MS analysis; however, pooling limits the ability to detect proteins that vary among individuals. In Experiment 2, some pooled replicates contained more males, increasing the risk that low‐abundance peptides were diluted below the MS detection threshold. We reduced potential individual‐level bias by using only 1 sample per male per replicate, ensuring no individuals disproportionately influenced the proteomic profile. This limits treatment comparisons to the level of the pooled sample rather than the individual, and observed differences in proteomic profiles should be interpreted as population‐level trends rather than individual‐level effects. In addition, males differed in age between the two experiments, which may also contribute to the small proportion of overlapping proteins. Older males may produce different relative abundances of specific proteins. Such biological variation as well as the constraints of pooled sampling likely reduced the consistency of protein detection across experiments.

## 5 Conclusion

In conclusion, we provide the first experimental evidence that pre‐ and postnatal developmental stress alters the proteome of a unique seminal foam produced by male Japanese quail, using an approach that is portable for work on seminal fluids across vertebrates. Our findings show that different types of early developmental stress exert a similar influence on the molecular composition of this seminal fluid, with potential long‐term impacts on ejaculate function and fertility. Contrary to our original hypothesis, developmentally stressed males do not reduce investment in SFP production, but instead we find evidence of oxidative stress and inflammation in their reproductive tissue with potential long‐term consequences for sperm function, future matings and other physiological functions. The elevated abundance of metabolic proteins that support sperm function in developmentally stressed males may reflect adaptive plasticity to safeguard fertilisation despite physiological constraints imposed by early‐life stress, potentially at the expense of future reproductive performance. Our findings provide new insights into mechanisms by which early‐life environmental conditions can influence reproductive fluids and the consequences of this for fertility in adulthood. Furthermore, they highlight the importance of developmental plasticity in modulating reproductive investment and function, potentially as an adaptive response to mitigate oxidative and inflammatory damage.

## Author Contributions

C.M. conceptualised the study, designed the methods, collected data, analysed the data, created the figures and wrote the manuscript; M.G. designed the methods and reviewed the manuscript; O.V. designed the methods, collected samples/data, and reviewed the manuscript; T.K.P. designed the methods, collected the data, analysed the data and reviewed the manuscript; R.G. contributed to the collection of data and reviewed the manuscript; B.T. designed the methods, collected samples and reviewed the manuscript; N.H. conceptualised the study, designed the methods and reviewed the manuscript.

## Funding

This work was supported by the Natural Environment Research Council (NE/S00713X/I), Deutsche Forschungsgemeinschaft (428800869), Schweizerischer Nationalfonds zur Förderung der Wissenschaftlichen Forschung (PP00P3 128386, PP00P3 157455), and Royal Society (DHF160200).

## Conflicts of Interest

The authors declare no conflicts of interest.

## Supporting information


**Data S1:** mec70257‐sup‐0001‐TablesS1‐S15.xlsx.

**Figure S1:** mec70257‐sup‐0002‐FigureS1.pdf.

## Data Availability

The mass spectrometry proteomics data have been deposited in the ProteomeXchange Consortium via the PRIDE (Perez‐Riverol et al. 2025) partner repository with the dataset identifier PXD063034.
