# Supplementary material for: Experimental Evidence That Prenatal and Postnatal Developmental Stress Affects the Adult Seminal Fluid Proteome in a Precocial Bird
Source: Mol Ecol. 2026 Jan 28;35(2):e70257. doi: 10.1111/mec.70257 (PMC12853081; doi:10.1111/mec.70257)
Supplement: Supplementary file 2 — Figure S1: mec70257‐sup‐0002‐FigureS1.pdf. [file MEC-35-e70257-s001.pdf]

MOLECULAR ECOLOGY

Supplemental Information for:

Experimental evidence that prenatal and postnatal developmental stress affects the adult seminal fluid proteome in a precocial bird.

Chloe Mason, Martin Garlovsky, Oscar Vedder, Trong Khoa Pham, Rachel George, Barbara Tschirren & Nicola Hemmings

Supplementary Figures

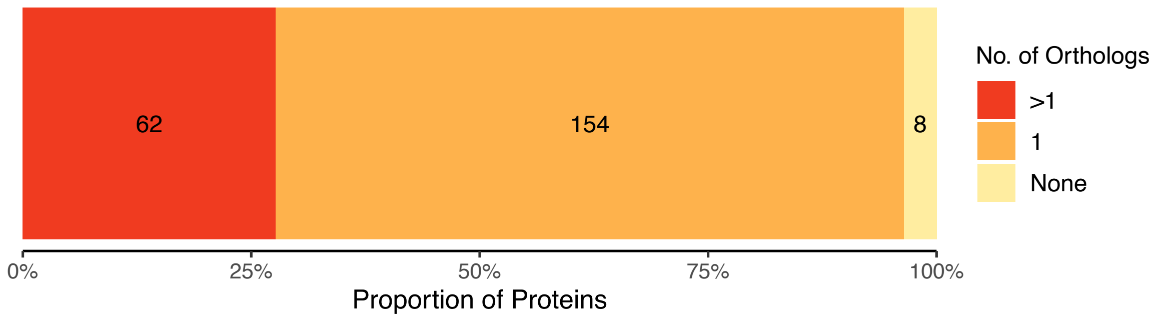

Figure S1. The proportion of Japanese quail (Coturnix japonica) seminal foam proteins that have more than one (red), one (orange), or no (yellow) chicken (Gallus gallus domesticus) orthologs.
